# Constraints on the source of ions in the Jianhe hot springs in Guizhou Province, China by water-rock interaction experiments

**DOI:** 10.1371/journal.pone.0324054

**Published:** 2025-06-18

**Authors:** Xiangheng Pu, Li Zhou, Zhengshan Chen, Wenge Zhou

**Affiliations:** 1 School of Geography and Environmental Science, Guizhou Normal University, Guiyang, China,; 2 State Key Laboratory Incubation Base for Karst Mountain Ecology Environment of Guizhou Province, Guiyang, China; 3 Key Laboratory of High-Temperature and High-Pressure Study of the Earth’s Interior, Institute of Geochemistry, Chinese Academy of Sciences, China; Yogi Vemana University, INDIA

## Abstract

Due to the lack of experimental studies, the effect of water-rock interactions on the hydrochemical characteristics of hot springs within belted reservoir remains poorly understood. To solve this issue, we analyzed the hydrochemical characteristics of the hot springs and the geochemical features of the reservoir rocks in the Jianhe hot springs in Guizhou province, SW China. All water sample analyses adhered to the China analytical procedures (GB 8538−2022), then carried out water-rock interacting experiments with representative reservoir rocks (e.g., metamorphosed tuff, metamorphosed quartz sandstone, and slate) under varying reaction time, temperature, and pH conditions. The results indicate that the concentration of dissolved ions in the solution increased with time, then gradually stabilized, reaching dynamic equilibrium around 35 days. Higher temperatures facilitated the leaching of K^+^, Na^+^, and H_2_SiO_3_, meanwhile reduced the leaching of Ca^2+^ and Mg^2+^. However, both Ca^2+^ and Mg^2+^ in the solution showed a pronounced response to pH changes from 4 to 10, whereas the K^+^, Na^+^, and H_2_SiO_3_ concentrations were less sensitive to pH changes. In particular, under experimental conditions corresponding to the reservoir (90°C), the Ca^2+^, concentrations as leached from metamorphosed tuff agreed well with the hydrochemical data in Jianhe hot springs, which are significantly lower than those in the solutions interacted with quartz sandstone or slate, and indicate that metamorphosed tuff should be the primary sources for K^+^, Na^+^, Ca^2+^, Mg^2+^ and H_2_SiO_3_ in the hot springs.

## Introduction

Water is a fundamental resource essential to life, sustaining ecosystems, and driving human civilization through its myriad uses [1–4]. Among its diverse forms, geothermal water, particularly from hot springs, stands out as a unique and valuable asset due to its elevated temperatures and enriched mineral content [5–7]. Hot springs formed in specific geological environments, such as fault zones, volcanic regions, and sedimentary basins, represent a significant geothermal resource [8–10] and are usually used in electricity generation, heating, and therapeutic applications [11,12]. The hydrochemical composition of the hot springs water is governed by the complex water-rock interaction processes under geothermal conditions [13–15]. Clarifying the sources of ions in the hot springs water and their enrichment processes is essential not only for revealing the formation mechanisms of hot springs but also for the utilization of the hot springs water [16–19].

Guizhou Province is located in the southwest of China, where large karst areas occur, and contains geothermal resources in karst reservoirs formed by carbonate erosion. The ionic composition of the hot springs water is dominated by Ca^2+^. In contrast, in the metamorphosed rocks area of southeastern Guizhou Province, there is a zonal reservoir controlled by water conduction along fault zone, and the cationic composition of hot springs water there is dominated by Na^+^. For example, Jianhe hot springs in southeastern Guizhou Province are controlled by the Gedong fault zone. The concentration of Na^+^ in Jianhe hot springs water is about 20 times that of the nearby Shiqian Chengbei hot springs [20]. The reservoir rocks of Jianhe hot springs are metamorphosed rocks, including metamorphosed tuff, metamorphosed siltstone, and metamorphosed sandstone [21]. Numerous studies have been conducted on Jianhe hot springs, including the determination of the content of major and trace elements in the hot springs water [22–25]. The results of previous studies indicated that the positive Eu anomaly in the hot springs water is caused by the decomposition of plagioclase [26]. Stable isotopic compositions of hydrogen and oxygen of the hot springs water suggested that atmospheric precipitation is the main source of water supply [27]. In addition, Chen [28] and Yang [29] demonstrated that the ion source of the Jianhe hot springs water is related to the reservoir rocks by comparing the chemical characteristics of the hot springs water with the geochemistry of reservoir rocks. Nevertheless, these field observations alone cannot completely clarify the constraints of different rock types on the ion source of the hot springs water.

Water-rock interaction simulation experiments are one of the important means to reveal the source of ions in hot water solution. Zhou et al. [30] explored the dissolution of granite and hot dry rock at different temperatures through water-rock interaction experiments. The results showed that the dissolution of Si and elements such as Na, K, Ca, and Al first increased and then decreased in the hot water solution. Huang et al. [31] explored the interaction between groundwater and sandstone reservoirs under recharge conditions. Their experiment effectively reproduced the water-rock interaction process and confirmed that the main dissolved minerals were dolomite and feldspar. Although numerous experimental studies on water-rock interactions have been conducted by previous researchers, investigations into the origins of ionic components in Jianhe hot springs remain confined to inferences drawn from hydrochemical characteristics [21]. While comparing the chemical properties of hot spring water with the geochemical features of reservoir rocks may suggest a connection between ion sources and the reservoir, this approach cannot directly constrain the specific origins of ions in Jianhe hot spring water.

In this paper, based on the analysis of chemical characteristics of hot springs water and geochemical characteristics of reservoir rocks of the Jianhe hot springs, we select representative reservoir rocks of the Jianhe hot springs to carry out water-rock interaction simulation experiments in which we have varied the interaction time, the temperature and pH. The aim of this study is to investigate the effect of reaction time, temperature, and pH on the ion concentration of the reaction solution, and further explore the control of the lithology on K^+^, Na^+^, Ca^2+^, Mg^2+^, and H_2_SiO_3_ in the Jianhe hot springs. Our experimental result will provide scientific evidence for the sustainable development and utilization of hot springs resources.

## Geological background of Jianhe hot springs

**Fig 1:**
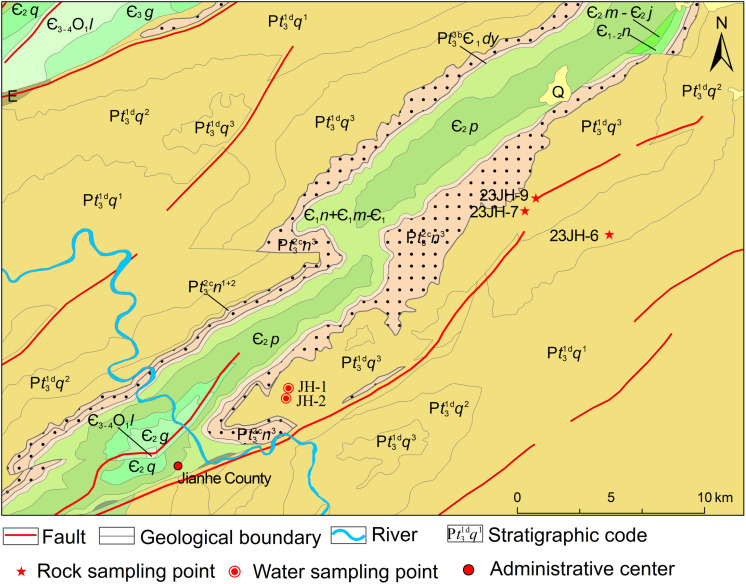
Simplified geological map of the study area (Adapted from Li [33]).

The Jianhe hot springs located at the southwestern edge of the South China Fold Belt. The emerge at the southwestern turning point of the Chongsuoxi Anticline and on the northwest side of the Gedong normal fault. The regional stratigraphy is composed of the Neoproterozoic Qingbaikou Formation and the Paleozoic Cambrian strata. These include the Qingshuijiang Formation, Nantuo Formation, and Dengying Formation of the Neoproterozoic Xiajiang Group, as well as the Cambrian Niutitang Formation, Jindingshan Formation, and Balang Formation (Fig 1). The exposed strata at the hot springs belong to the Qingshuijiang Formation, and the lithology primarily consists of metamorphosed rocks, including metamorphosed tuff, metamorphosed siltstone, metamorphosed sandstone, and slate [32]. The formation of the hot springs is controlled by the Gedong Fault, a normal fault with a strike of 50° ~ 60° and a dip angle of about 50°. The footwall consists of the Qingshuijiang Formation and Pinglue Formation of the Xiajiang Group, while the hanging wall ranges from the Pinglue Formation to the Lower Cambrian Jiumenchong Formation (Fig 1). The Gedong Fault formed during the Xuefeng orogeny and was reactivated during the Caledonian, Yanshan, and Himalayan orogenies. Mafic and ultramafic rock bodies are exposed along the fault zone. The fault zone depicts a significant geothermal and hydrothermal structure in the region.

The Jianhe hot springs is controlled by the Gedong Fault zone, and is classified as a belted reservoir, a geothermal reservoir where effective porosity and permeability are distributed in a belt-like pattern.Its water source is primarily recharged by atmospheric precipitation. Under the influence of gravity, precipitation moves downward through structural channels, where it is heated by regional geothermal flow conducted along the fault, undergoing deep circulation (about 2890m) [21]. The heated and expanded thermal fluid rises due to buoyancy, interacting with shallow low-temperature fluids through convective circulation. The water flows along structural fissures and, in regions insulated by impermeable metamorphosed rocks, rapidly ascends through fractures that act as conduits for thermal groundwater, ultimately forming hot springs at the surface.

## Sample collection and analysis

In this study, two water samples and three rock samples were collected from the Jianhe hot springs (Fig 1). Clean, uncontaminated 1500 ml polyethylene bottles were used for water sampling. Prior to sampling, the bottles were rinsed with distilled water. During sampling, the hot springs water was filtered using a 45 μm needle-type PES (polyethersulfone) filter. The filtered water was then used to rinse the sampling bottles three times before the water sample was slowly poured into the bottles until overflowing, after which the caps were securely tightened to complete the collection. Simultaneously, a portable water quality analyzer (Multi 360 IDS) manufactured by WTW (Germany) was used on-site to measure the temperature, pH, and TDS (total dissolved solids) of the water samples. The rock sampling sites were situated near the boundary between Jianhe County and Sansui County in Guizhou Province (Fig 1), near the Gedong Fault. The collected rock samples (Fig 1) included metamorphosed quartz sandstone (23JH-7), slate (23JH-9), and metamorphosed tuff (23JH-6) from the surrounding rocks of the Jianhe hot springs reservoir.

The concentrations of K^+^, Na^+^, Ca^2+^, and Mg^2+^ in the hot spring water were analyzed using a Vista MPX ICP-OES from Agilent. Cl^−^, F^−^, and SO_4_^2−^ were tested using an ICS-90 ion chromatograph from DIONEX, while HCO_3_^−^ was determined using a titration method. The H_2_SiO_3_ concentrations in both hot springs water and experimental water samples were quantified using a TU-1901 UV-visible spectrophotometer. For the experimental water samples, K^+^, Na^+^, Ca^2+^, and Mg^2+^ concentrations were measured using an AA-7000 atomic absorption spectrometer from Shimadzu. All water sample analyses adhered to the China analytical procedures [34]. The major element compositions of the rock samples were determined using an ARL Perform’X 4200 X-ray fluorescence spectrometer from Thermo Fisher, and the mineralogical composition was analyzed using a D8 ADVANCE X-ray diffractometer from Bruker.

## Water-rock interaction experiments

### Starting materials

The rock samples used in this experiment included metamorphosed tuff from the second section of the Qingshuijiang Formation and metamorphosed quartz sandstone and slate from the third section of the same formation. Fresh rock samples were initially broken into smaller pieces using a hammer wrapped in coarse cloth. Subsequently, in order to complete the experiment in a short time, they were further crushed to a particle size of 200 mesh using a planetary agate mill, without cleaning to preserve their original geochemical properties. The reaction solutions consisted of aqueous solutions with pH values of 4, 7 (pure water), and up to pH 10. The pH was adjusted using HCl and NaOH solutions and measured with a Pius TM FE28 pH meter from Mettler Toledo.

### Experimental methods

The experiments were carried out in polypropylene (PP) reaction bottles, and the bottlenecks were sealed with soft silicone gaskets to prevent water vapor loss. A water-to-rock mass ratio of 20:1 was used to weigh the corresponding amount of rock samples (50g), which were then placed into the PP bottles, followed by the addition of 1000 ml of aqueous solution. The heating was conducted using an electric thermostatic forced-air drying oven, with temperature measured by the oven’s built-in thermometer, achieving a temperature measurement accuracy of 0.1°C and a temperature fluctuation of ± 0.2°C during the experiments. At regular intervals (see Table 1), approximately 5 ml of clear supernatant was carefully withdrawn from the top layer using a syringe, filtered through a 0.45 μm membrane, and then collected into clean PP bottles. Whereafter, an equivalent amount of fresh solution was added to the reaction apparatus to compensate for the sampled volume. The bottles were then shaken to mix the rock and solution before being placed back into the thermostatic drying oven for continued heating. A total of 15 water-rock interaction simulation experiments were conducted using the different rock types, different temperatures and pH conditions. The extracted water samples were analyzed for major ion components and metasilicic acid. Detailed experimental conditions are listed in Table 1.

**Table 1:** Overview of experimental conditions.

Run Number	Rock Sample	Solution System	Temperature (°C)	Reaction Time (days)
WR6−1	Metamorphosed tuff	pH = 7 (pure Water)	25 °C	1, 3, 7, 14, 21, 28, 35, 42, 49, 56, 63, 70, 77, 84, and 91
WR6−2			60 °C	
WR6−3			90 °C	
WR6−4		pH = 4 (HCl dilute solution)	60 °C	
WR6−5		pH = 10 (NaOH dilute solution)	60 °C	
WR6−6	Metamorphosed quartz sandstone	pH = 7 (pure Water)	25 °C	
WR6–7			60 °C	
WR6–8			90 °C	
WR6–9		pH = 4 (HCl dilute solution)	60 °C	
WR6–10		pH = 10 (NaOH dilute solution)	60 °C	
WR6–11	Slate	pH = 7 (pure Water)	25 °C	
WR6–12			60 °C	
WR6–13			90 °C	
WR6–14		pH = 4 ((HCl dilute solution)	60 °C	
WR6–15		pH = 10 (NaOH dilute solution)	60 °C	

## Results

### Hydrochemical characteristics of Jianhe hot springs

**Fig 2:**
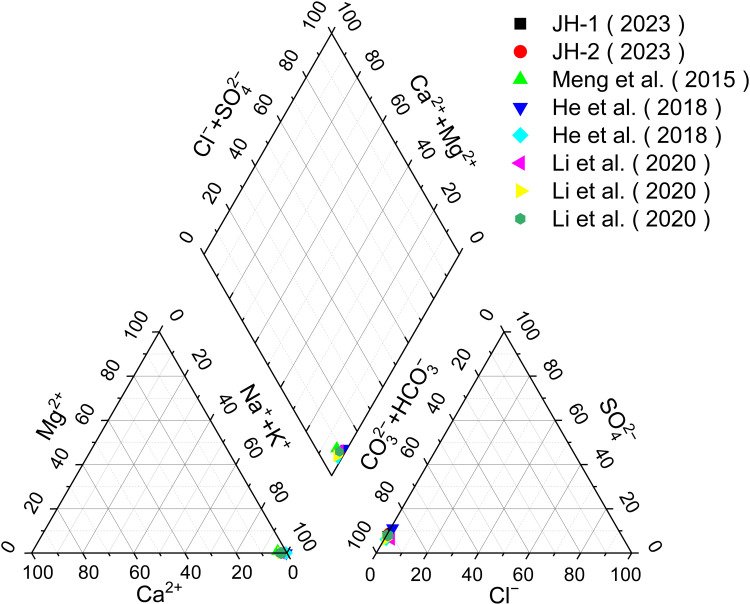
Piper diagram of water samples from Jianhe hot springs [21,26,35].

The analysis of the Jianhe hot springs water samples, JH-1 and JH-2, indicates that their chemical properties are similar. A comparison with previous analytical results (Table 2) shows that the outlet water temperature of Jianhe hot springs remains between 40.1°C and 49.6 °C year-round, with a pH range of 8.00 to 8.94, and TDS between 337.00 and 419.00 mg/L. The primary cation is Na^+^, with concentrations ranging from 85.1 to 118.00 mg/L, while the concentrations of Ca^2+^ (0.30 ~ 2.90 mg/L), Mg^2+^ (0.01 ~ 0.75 mg/L), and K^+^ (1.7 ~ 8.69 mg/L) are relatively low.The dominant anion is HCO_3_^−^ (176.28 ~ 226.00 mg/L), followed by Cl^−^ and SO_4_^2−^ (0.96 ~ 5.31 mg/L and 12 ~ 28.60 mg/L, respectively). Additionally, the concentration of H_2_SiO_3_ in the hot springs water is between 55.71 and 62.67 mg/L. According to the water chemistry trilinear diagram (Fig 2), the hydrochemical type of Jianhe hot springs is classified as HCO_3_^−^–Na water.

**Table 2:** The hydrochemical components of Jianhe hot springs from 2015 to 2023.

Sample	Primary cation/(mg/L)				Main anion/(mg/L)				H_2_SiO_3_	TDS	Data year	Data source
**K** ^**+**^	**Na** ^**+**^	**Ca** ^**2+**^	**Mg** ^**2+**^	**F** ^**−**^	**Cl** ^**−**^	**HCO**_**3**_^**−**^	**SO**_**4**_^**2−**^				
Jianhe hot springs	2.53	108.97	1.45	0.06	1.5	1.01	195.1	21.35	58.83	419	2023	This study
	2.55	108.44	1.14	0.03	1.59	0.84	185.6	17.67	62.67	409		
	1.88	85.1	2.9	0.75	1.68	1.65	223.19	18.19	60.47	337	2015	[21]
	3.29	118	0.3	—	2.42	1.97	224	28.6	—	—	2018	[26]
	8.69	111	0.51	—	3.86	1.84	226	15.1	—	—		
	1.7	95	2.33	0.03	2	5.31	176.28	12	55.71	343.13	2020	[35]
	1.8	95	2.33	0.03	2.2	0.96	182.58	12	56.27	343.79		
	1.8	98	2.33	0.01	2	0.96	182.58	16	55.71	352.66		
Average (SD)	3.03 (2.35)	102.44 (10.86)	1.66 (0.95)	0.15 (0.29)	2.16 (0.76)	1.81 (1.48)	199.42 (21.34)	17.61 (5.44)	58.28 (2.89)	367.43 (36.55)		

“—” indicates “not detected”.

### Geochemical characteristics of the Jianhe hot springs reservoir rocks

**Fig 3. X:**
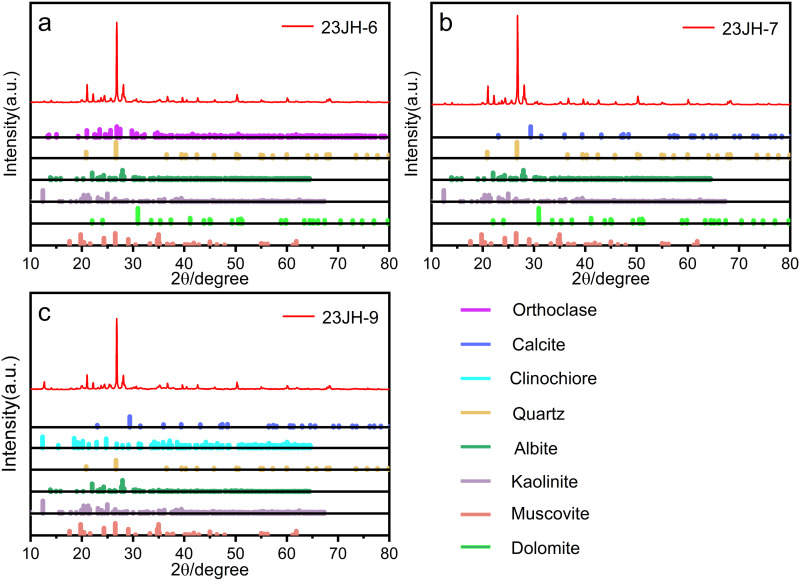
ray diffraction (XRD) pattern of rock sample.

The mineral compositions of three metamorphosed rock samples were determined through XRD analysis (Fig 3). The metamorphosed tuff (23JH-6) consists predominantly of quartz, orthoclase, albite, muscovite, kaolinite, and dolomite. The metamorphosed quartz sandstone (23JH-7) consists predominantly of quartz, albite, muscovite, kaolinite, dolomite, and calcite, while the slate (23JH-9) consists predominantly of quartz, albite, muscovite, calcite, kaolinite, and chlorite.

Table 3 shows that the SiO_2_ content in samples 23JH-6 (metamorphosed tuff), 23JH-7 (metamorphosed quartz sandstone), and 23JH-9 (slate) is 71.56 wt %, 75.87 wt %, and 63.94 wt %, respectively. Al_2_O_3_ and Fe_2_O_3_ are significant indicators of rock chemical composition, with Al_2_O_3_ contents of 13.75 wt %, 11.82 wt %, and 17.33 wt % in samples 23JH-6, 23JH-7, and 23JH-9, respectively. The highest Al_2_O_3_ content is found in sample 23JH-9, together with the highest Fe_2_O_3_ content of 5.60 wt %. The contents of Na_2_O and K_2_O in the rock samples are also relatively high, with Na_2_O content being generally higher than that of K_2_O. Sample 23JH-7 has the highest Na_2_O (4.2 wt %) and K_2_O (2.6 wt %) contents. Additionally, the MgO (1.37 wt %) and CaO (0.88 wt %) contents in sample 23JH-9 are also relatively high.

**Table 3:** Chemical composition of Jianhe hot springs reservoir rocks.

Sample number (Rock type)	Composition (wt %)											
**SiO**_**2**_	**Al**_**2**_ **O**_**3**_	**Fe**_**2**_ **O**_**3**_	**MgO**	**CaO**	**Na**_**2**_ **O**	**K**_**2**_ **O**	**MnO**	**P**_**2**_ **O**_**5**_	**TiO**_**2**_	**LOI**	**SUM**
23JH-6 (metamorphosed tuff)	71.56	13.75	3.69	0.52	0.39	3.68	2.32	0.09	0.05	0.63	2.50	99.18
23JH-7 (metamorphosed quartz sandstone)	75.87	11.82	1.54	0.57	1.87	4.20	1.03	0.10	0.06	0.33	1.83	99.22
23JH-9 (slate)	63.94	17.33	5.60	1.37	0.88	2.99	2.60	0.14	0.11	0.76	3.41	99.13

### Water-rock interaction experimental results

#### Variation of dissolved ion concentrations with reaction time.

**Fig 4:**
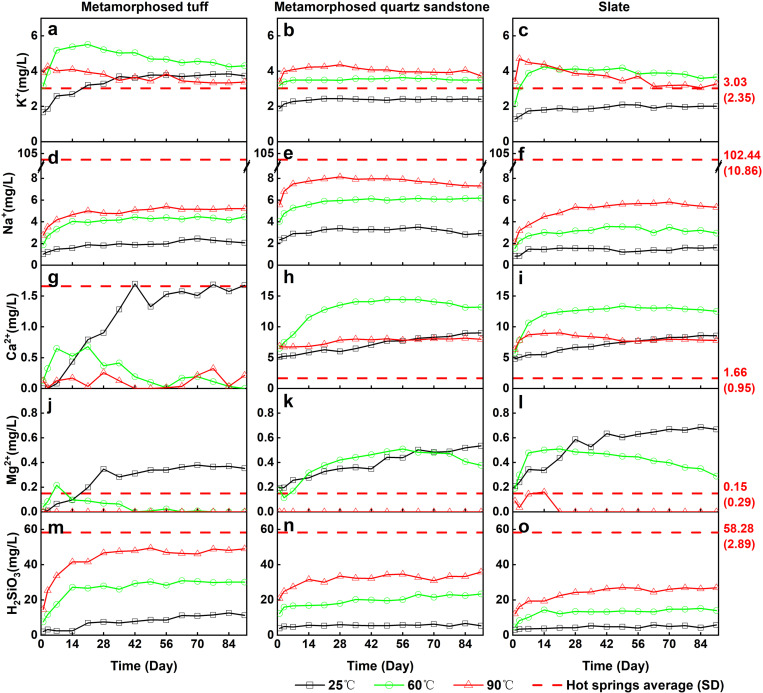
Temperature-dependent changes in the concentrations of major components.

**Fig 5:**
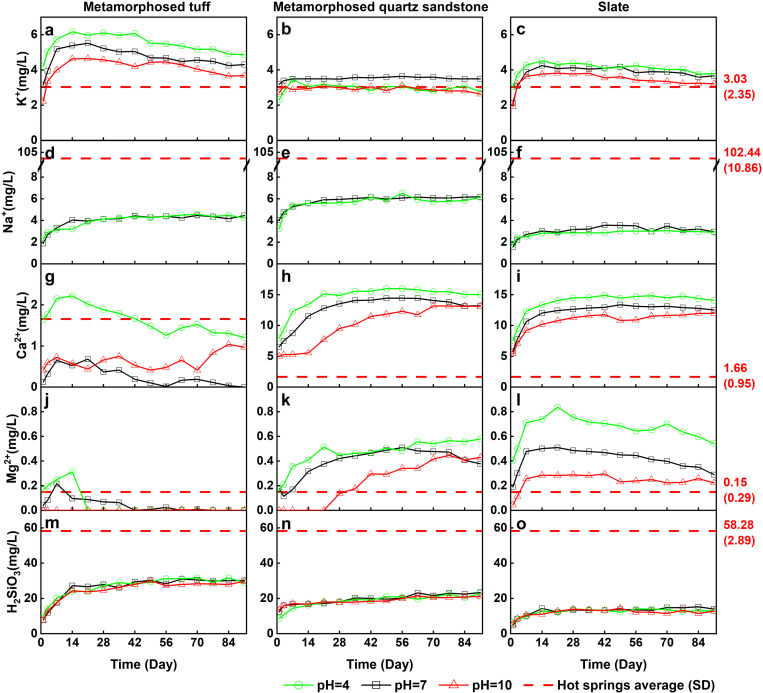
pH-dependent changes in the concentrations of major components.

The concentration of major components in the experimental solution exhibited significant changes over time (Fig 4 and 5), and the experiments results are shown in Appendix table 1. The concentrations of K^+^, Na^+^, and H_2_SiO_3_ initially increased rapidly. After about 28 days Na^+^ and H_2_SiO_3_ showed a trend towards stabilization. The K^+^ concentration exhibited both stabilization and a slow decreasing trend in different lithologies. In contrast, the concentrations of Ca^2+^ and Mg^2+^ continued to increase at 25°C, while at 60°C and 90°C, their concentrations displayed a slight decrease or minor fluctuations. After 35 days, the reaction tended toward stability, with the concentrations of K^+^, Na^+^, and H_2_SiO_3_ showing minimal changes. The behavior of Ca^2+^ and Mg^2+^ was more complex, exhibiting phenomena such as gradual decrease, gradual increase, stabilization, rapid increase, and rapid decrease.

#### Variation of dissolved ion concentrations with temperature.

The trends in the concentrations of major components in water under different temperature conditions exhibited significant differences (Fig 4). The concentrations of Na^+^ and H_2_SiO_3_ increased with rising temperature, demonstrating a clear positive correlation. Conversely, K^+^, Ca^2+^, and Mg^2+^ exhibited a negative correlation with increasing temperature. For instance, after 35 days of experimentation with the metamorphosed tuff, the concentrations of K^+^, Na^+^, and H_2_SiO_3_ were in the ranges of 3.61 to 3.85 mg/L, 1.86 to 2.45 mg/L, and 7.8 to 12.7 mg/L, respectively, at 25°C. At 60°C, the concentrations increased to 4.25 to 5.05 mg/L for K^+^, 4.16 to 4.45 mg/L for Na^+^, and 28.34 to 30.94 mg/L for H_2_SiO_3_, representing 1.29 times, 1.99 times, and 2.88 times the respective values at 25°C. Meanwhile, at 90°C, the concentrations were 3.44 to 3.85 mg/L for K^+^, 4.76 to 5.39 mg/L for Na^+^, and 46.15 to 49.03 mg/L for H_2_SiO_3_, corresponding to 0.97 times, 2.35 times, and 4.64 times the concentrations at 25°C.

### Variation of dissolved ion concentrations with pH

The influence of pH variation on the dissolution of Na^+^, K^+^, and H_2_SiO_3_ is relatively minor, while it significantly affects the dissolution of Ca^2+^ and Mg^2+^ (Fig 5). After 35 days of reaction, the concentration of K^+^ under pH = 10 was generally lower than that observed at pH = 7. Under pH = 4 conditions, the concentrations of K^+^ in the metamorphosed tuff and slate samples were slightly higher than those at pH = 7, whereas the concentration of K^+^ in the metamorphosed quartz sandstone sample was comparable to that at pH = 10. In contrast, the concentrations of Ca^2+^ and Mg^2+^ at pH = 4 were higher than those observed at pH = 7 and pH = 10. The response of H_2_SiO_3_ to pH variation was minimal, making it difficult to clearly discern its effects.

## Discussion

### Main controlling factors on the dissolved ion concentrations

**Fig 6:**
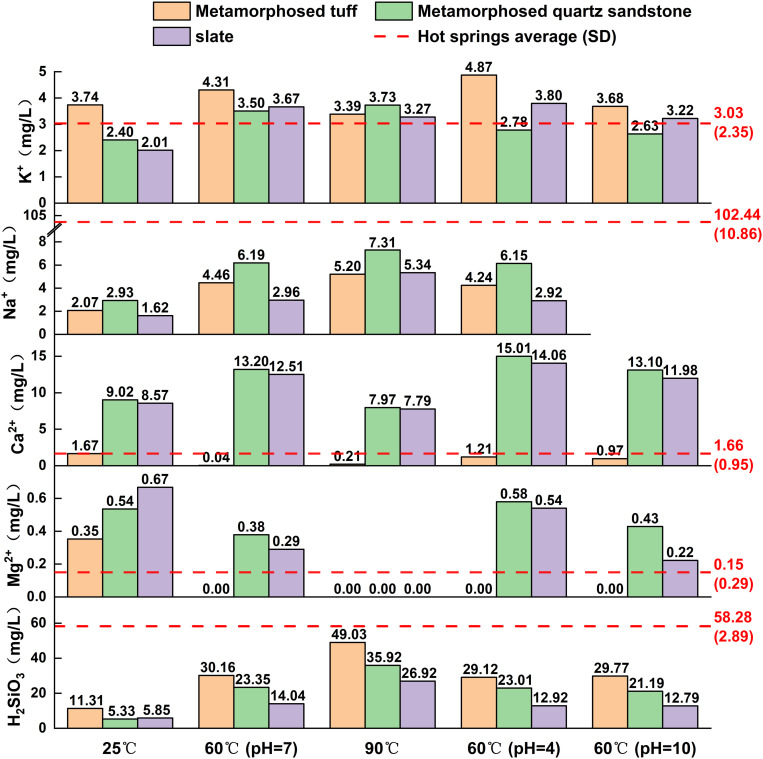
Concentrations of major Ions under different conditions on day 91.

The concentrations of dissolved ions during water-rock interactions are primarily governed by reaction time, temperature, pH, and lithology. In this study, while not all reactions reached equilibrium after 91 days, the majority appeared to stabilize, thus, these results were approximated as equilibrium conditions for plotting purposes. The dissolved ion concentrations from the final sampling (Day 91) and their variations with lithology are shown in Fig 6. The following sections elaborate on the behavior of ions under these factors.

#### Release of K^+^.

The concentration of K^+^ exhibits varying responses to reaction time, temperature, pH, and lithology, resulting in an overall complex behavior. Over the course of the reaction, K^+^ concentrations in the metamorphosed tuff and slate groups initially increase rapidly before gradually declining, whereas in the metamorphosed quartz sandstone group, K^+^ concentrations rise quickly and then stabilize. This pattern may be attributed to ion exchange processes. Ion exchange is a physicochemical process in which one ion is removed from the solution and replaced by another ion, usually occurring on the surface of solids. Clay minerals possess excellent ion-exchange properties, with their exchange capacity being greater at 80°C than at room temperature [36]. A similar phenomenon was observed in this study: at 90°C, the K^+^ concentrations in the metamorphosed tuff and slate groups were lower in the later stages of the experiment than at 60°C (Fig 6). The negatively charged surface of clay minerals can adsorb positively charged ions. When both K^+^ and Na^+^ are present, they are easily adsorbed onto the solid surface; however, due to their different hydration radii, K^+^ is more likely to replace Na^+^, leading to a decrease in K^+^ concentration in the solution. During prolonged leaching, K^+^ gradually replaces Na^+^, resulting in higher Na^+^ concentrations than K^+^ concentrations in the hot springs water. This effect is less pronounced in the metamorphosed quartz sandstone group, likely due to a lower abundance of clay minerals compared to the metamorphosed tuff and slate groups. With increasing pH, the K^+^ concentrations in both the metamorphosed tuff and slate groups exhibit a consistent decline, while the metamorphosed quartz sandstone group shows a slight suppression under both acidic and alkaline conditions (Fig 6). As temperature rises, the K^+^ concentrations in the metamorphosed tuff and slate groups initially increase before decreasing, whereas the metamorphosed quartz sandstone group displays a gradual upward trend.

#### Release of Na^+^.

The leaching of Na^+^ exhibits a trend of rapid increase followed by stabilization as reaction time progresses. After 35 days, the Na^+^ concentration under all conditions shows minimal variation with time, indicating that a stable state has largely been achieved. The leaching of Na^+^ is notably responsive to temperature, displaying a positive correlation with rising temperature. At 90°C, the Na^+^ concentration reaches its highest value, suggesting that elevated temperatures enhance Na^+^ leaching. Due to the use of NaOH to adjust alkaline conditions (pH = 10), the leaching of Na^+^ under alkaline conditions is not discussed here. Under acidic conditions (pH = 4), the Na^+^ concentration is comparable to that at pH = 7. This indicates that a decrease in pH during the experiment has a negligible impact on Na^+^ concentrations. Among the different lithologies, the metamorphosed quartz sandstone group exhibits the highest Na^+^ leaching, indicating a potentially greater abundance of Na-bearing minerals in this rock type. This is supported by XRF analysis, which reveals the highest Na_2_O content in the metamorphosed quartz sandstone (Table 3).

#### Release of Ca^2+^.

The leaching of Ca^2+^ is significantly influenced by lithological variations, with the metamorphosed quartz sandstone and slate groups displaying comparable Ca^2+^ concentrations that are substantially higher than those in the metamorphosed tuff group. This difference may be attributed to the types and abundances of Ca-bearing minerals in the rocks. XRD analysis reveals that the primary Ca-bearing mineral in the metamorphosed tuff is dolomite, whereas both dolomite and calcite are present in the metamorphosed quartz sandstone and slate. Additionally, XRF results indicate that the CaO content in the metamorphosed tuff is the lowest among the three lithologies. Previous studies have shown that calcite dissolves more readily than dolomite, which likely explains why the Ca^2+^ concentration in the metamorphosed tuff group is substantially lower than in the quartz sandstone and slate groups. Due to the overall low Ca^2+^ concentrations in the metamorphosed tuff, measurement errors from the instrument may have a more pronounced impact when comparing leaching results across different conditions. Therefore, discussions on the effects of time, temperature, and pH primarily focus on the quartz sandstone and slate groups. With increasing reaction time, the Ca^2+^ concentrations in the quartz sandstone and slate groups, except at 25°C, exhibit a rapid increase in the early stages, stabilizing after 35 days. At 25°C, the slower reaction rate suggests that Ca^2+^ concentrations may require a longer duration to reach equilibrium. As reaction temperature increases, Ca^2+^ concentrations initially rise and then decline, peaking around 60°C, a trend consistent with findings from previous studies. Meanwhile, an increase in pH appears to reduce Ca^2+^ leaching to some extent.

#### Release of Mg^2+^.

The leaching of Mg^2+^ is significantly influenced by temperature variations, exhibiting a negative correlation with increasing temperature. The phenomenon of inverse solubility is frequently observed in systems such as magnesium sulfate and magnesium carbonate [37]. Combined with XRD analysis results, this indicates that the magnesium in the water primarily originates from dolomite. Across all lithological groups, the highest Mg^2+^ concentrations are observed at 25°C, while the lowest occur at 90°C. The metamorphosed tuff group displays the lowest overall Mg^2+^ concentrations, with minimal leaching at 60°C, whereas the metamorphosed quartz sandstone and slate groups still show slight leaching at this temperature. XRF analysis reveals that the MgO content in the metamorphosed tuff is the lowest among the three lithologies. The effect of pH on Mg^2+^ leaching is relatively minor: at pH = 4, a slight enhancement is observed, while at pH = 10, Mg^2+^ leaching either decreases marginally or remains largely unaffected. With increasing reaction time, Mg^2+^ concentrations generally stabilize after approximately 35 days.

#### Release of H_2_SiO_3_.

The leaching of H_2_SiO_3_ is significantly influenced by temperature, exhibiting a strong positive correlation with increasing temperature. For instance, in the metamorphosed quartz sandstone group, the H_2_SiO_3_ concentration at 90°C reaches a peak of 35.92 mg/L, which is 1.54 times and 6.74 times higher than the concentrations at 60°C and 25°C, respectively. With increasing reaction time, H_2_SiO_3_ concentrations rise rapidly in the early stages, stabilizing after 35 days. The leaching of H_2_SiO_3_ is also markedly affected by lithology. At 90°C, the metamorphosed tuff group records the highest H_2_SiO_3_ concentration of 49.03 mg/L, which is 1.37 times and 1.82 times greater than that of the metamorphosed quartz sandstone and slate groups, respectively, under the same conditions. In contrast, the influence of pH on H_2_SiO_3_ leaching is relatively minor, as both the leaching trends over time and the concentrations approaching equilibrium do not differ significantly across different pH levels.

### Ions source of Jianhe hot springs

Previous studies indicate that the Jianhe hot springs water primarily originates from atmospheric precipitation, with its recharge area located in the northern mountainous region [21]. For hot springs water is recharged by atmospheric precipitation, and the main source of ions is derived from water-rock interactions [38]. Regarding the sources of components in hot spring water, previous studies have typically analyzed the water’s composition and directly inferred potential origins. For instance, Meng et al. [21] measured the Ca^2+^ concentration in Jianhe hot springs and suggested that it is controlled by dolomite and calcite. However, such inferences may appear somewhat arbitrary and oversimplified. In this study, we found that the average Ca^2+^ concentration in Jianhe hot springs is 1.66 mg/L, a value closely matched by the results from the metamorphosed tuff group, while the Ca^2+^ concentrations in the metamorphosed quartz sandstone and slate groups are significantly higher, and Mg^2+^ exhibits a similar phenomenon. The Ca^2+^ leaching from metamorphosed quartz sandstone and slate is 4.80 and 4.69 times higher than the Ca^2+^ concentration in Jianhe hot springs water. This suggests that the primary source of Ca^2+^ in Jianhe hot spring water is likely the metamorphosed tuff. A comparison of the XRD results for the three rock types reveals that calcite is a major mineral constituent in both the metamorphosed quartz sandstone and slate, whereas the only Ca-bearing mineral in the metamorphosed tuff is dolomite. Therefore, the Ca^2+^ in Jianhe hot springs is likely derived from the dissolution of dolomite in the metamorphosed tuff, rather than calcite. Having established that the components in the hot springs predominantly originate from the metamorphosed tuff based on Ca^2+^, the sources of K^+^, Na^+^, Mg^2+^, and H_2_SiO_3_ can be readily deduced. In addition, by comparing the water chemistry characteristics with the XRD results of the metamorphosed tuff, K^+^ and Na^+^ are likely primarily leached from orthoclase, muscovite, and albite in the metamorphosed tuff, Mg^2+^ is derived from dolomite, and H_2_SiO_3_ originates from the leaching of orthoclase, albite, muscovite, quartz, and kaolinite. The concentrations of K^+^ and H_2_SiO_3_ in Jianhe hot spring water are 3.03 2.35 mg/L and 58.28 2.89 mg/L, respectively, which are also close to the dissolution amounts of the metamorphosed tuff group (90°C), with values of 3.39 mg/L for K^+^ and 49.03 mg/L for H_2_SiO_3_. This further suggests that metamorphosed tuff may be one of the primary rocks involved in the water-rock interaction under thermal reservoir conditions.

However, the Na^+^ concentration in Jianhe hot springs water is relatively high, being 20.7 times higher than the Na^+^ concentration in the metamorphosed tuff group (90°C) experiment. The concentration of Cl^−^ in Jianhe hot springs is relatively low, and since Cl^−^ is unlikely to precipitate during water-rock interactions, the dissolution of halite from the rock strata or the infiltration of surface water can be preliminarily ruled out as potential sources. Therefore, the low Na^+^ concentration in the metamorphosed tuff group may be related to the water to rock ratio involved in the reaction. For example, Zhang et al. [39] conducted water-rock interaction experiments with a lower water-rock mass ratio (1:2), and the Na^+^ concentration in the solution reached 400 mg/L after the experiment. In contrast, in this study, a higher water-rock mass ratio (20:1) was used, resulting in less Na^+^ dissolution in the water.

## Conclusions

In this study, water-rock interaction experiments were conducted under varying durations, temperatures, and pH conditions using metamorphosed tuff, metamorphosed quartz sandstone, and slate from the reservoir rocks of Jianhe hot springs. The conclusions are as follows:

(1) The water-rock interaction reaches dynamic equilibrium after approximately 35 days, with variations influenced by temperature and pH. An increase in temperature promotes the dissolution of K^+^, Na^+^, and H_2_SiO_3_, while inhibiting the dissolution of Ca^2+^ and Mg^2+^. Within the pH range of 4–10, pH changes have a relatively small effect on H_2_SiO_3_ dissolution but a significant impact on the dissolution of K^+^, Na^+^, Ca^2+^, and Mg^2+^. As pH increases, the dissolution of K^+^, Ca^2+^, and Mg^2+^ is inhibited.
(2) The water-rock interaction experiments under reservoir conditions (90°C) show that the Ca^2+^ leaching from metamorphosed quartz sandstone and slate is 4.80 and 4.69 times higher than the Ca^2+^ concentration in Jianhe hot springs water, respectively. In contrast, the Ca^2+^ dissolution from the metamorphosed tuff group is similar to the concentration in the hot springs water. Metamorphosed tuff is one of the primary rocks involved in the water-rock interaction under reservoir conditions, which constrains the concentrations of Ca^2+^, K^+^, H_2_SiO_3_, and even Na^+^ in Jianhe hot springs water.

Specifically, our results elucidate the dissolution characteristics of different lithologies in water-rock interactions, offering a scientific basis for evaluating changes in hot spring water chemistry. Furthermore, through this study, we have determined that the chemical composition of Jianhe hot spring water primarily originates from the metamorphosed tuff of the Qingshuijiang Formation, providing valuable practical data for hot spring development.

## Supporting information

S1 Table Experimental results of water-rock interaction. (PDF)
