# Supplementary material for: Constraints on the source of ions in the Jianhe hot springs in Guizhou Province, China by water-rock interaction experiments
Source: PLoS One. 2025 Jun 18;20(6):e0324054. doi: 10.1371/journal.pone.0324054 (PMC12176157; doi:10.1371/journal.pone.0324054)
Supplement: S1 Table — (PDF) [file pone.0324054.s001.pdf]

Appendix table 1 Experimental results of water-rock interaction

Sample Number	Rock Type	Solution System	temperature	time	Na^+	K^+	Ca^{2+}	Mg^{2+}	H_2SiO_3
			℃	days	mg/L				
WR6-1-1	metamorphosed tuff	pH = 7 (pure water)	25	1	1.02	1.66	0.00	0.00	2.08
WR6-1-2	metamorphosed tuff	pH = 7 (pure water)	25	3	1.22	1.85	0.00	0.01	3.25
WR6-1-3	metamorphosed tuff	pH = 7 (pure water)	25	7	1.47	2.60	0.08	0.07	2.47
WR6-1-4	metamorphosed tuff	pH = 7 (pure water)	25	14	1.57	2.69	0.44	0.10	2.47
WR6-1-5	metamorphosed tuff	pH = 7 (pure water)	25	21	1.87	3.21	0.79	0.20	7.02
WR6-1-6	metamorphosed tuff	pH = 7 (pure water)	25	28	1.81	3.28	0.90	0.35	7.54
WR6-1-7	metamorphosed tuff	pH = 7 (pure water)	25	35	1.97	3.70	1.28	0.28	7.02
WR6-1-8	metamorphosed tuff	pH = 7 (pure water)	25	42	1.87	3.61	1.70	0.31	7.80
WR6-1-9	metamorphosed tuff	pH = 7 (pure water)	25	49	1.92	3.78	1.33	0.34	8.71
WR6-1-10	metamorphosed tuff	pH = 7 (pure water)	25	56	1.96	3.77	1.53	0.34	8.58
WR6-1-11	metamorphosed tuff	pH = 7 (pure water)	25	63	2.30	3.70	1.57	0.36	11.18
WR6-1-12	metamorphosed tuff	pH = 7 (pure water)	25	70	2.45	3.76	1.51	0.38	10.92
WR6-1-13	metamorphosed tuff	pH = 7 (pure water)	25	77	2.29	3.82	1.68	0.37	11.57
WR6-1-14	metamorphosed tuff	pH = 7 (pure water)	25	84	2.17	3.84	1.57	0.37	12.61
WR6-1-15	metamorphosed tuff	pH = 7 (pure water)	25	91	2.07	3.74	1.67	0.35	11.31
WR6-2-1	metamorphosed tuff	pH = 7 (pure water)	60	1	1.87	3.17	0.12	0.04	7.54
WR6-2-2	metamorphosed tuff	pH = 7 (pure water)	60	3	2.69	3.95	0.33	0.08	11.70
WR6-2-3	metamorphosed tuff	pH = 7 (pure water)	60	7	3.32	5.19	0.65	0.22	17.42

Sample Number	Rock Type	Solution System	temperature	time	Na^+	K^+	Ca^{2+}	Mg^{2+}	H_2SiO_3
			℃	days	mg/L				
WR6-2-4	metamorphosed tuff	pH = 7 (pure water)	60	14	4.04	5.38	0.53	0.10	27.30
WR6-2-5	metamorphosed tuff	pH = 7 (pure water)	60	21	3.95	5.52	0.68	0.09	26.65
WR6-2-6	metamorphosed tuff	pH = 7 (pure water)	60	28	4.12	5.23	0.37	0.07	27.95
WR6-2-7	metamorphosed tuff	pH = 7 (pure water)	60	35	4.16	5.04	0.41	0.06	26.13
WR6-2-8	metamorphosed tuff	pH = 7 (pure water)	60	42	4.43	5.05	0.19	0.00	29.38
WR6-2-9	metamorphosed tuff	pH = 7 (pure water)	60	49	4.28	4.69	0.10	0.01	30.29
WR6-2-10	metamorphosed tuff	pH = 7 (pure water)	60	56	4.39	4.68	0.01	0.02	28.34
WR6-2-11	metamorphosed tuff	pH = 7 (pure water)	60	63	4.25	4.48	0.17	0.00	30.94
WR6-2-12	metamorphosed tuff	pH = 7 (pure water)	60	70	4.47	4.56	0.19	0.01	30.55
WR6-2-13	metamorphosed tuff	pH = 7 (pure water)	60	77	4.35	4.49	0.11	0.00	29.90
WR6-2-14	metamorphosed tuff	pH = 7 (pure water)	60	84	4.17	4.25	0.04	0.00	30.16
WR6-2-15	metamorphosed tuff	pH = 7 (pure water)	60	91	4.46	4.31	0.04	0.00	30.16
WR6-3-1	metamorphosed tuff	pH = 7 (pure water)	90	1	2.77	3.91	0.12	0.00	14.69
WR6-3-2	metamorphosed tuff	pH = 7 (pure water)	90	3	3.52	4.26	0.01	0.00	25.35
WR6-3-3	metamorphosed tuff	pH = 7 (pure water)	90	7	4.17	4.02	0.12	0.00	33.80
WR6-3-4	metamorphosed tuff	pH = 7 (pure water)	90	14	4.66	4.10	0.17	0.00	41.60
WR6-3-5	metamorphosed tuff	pH = 7 (pure water)	90	21	5.00	3.94	0.04	0.00	41.60
WR6-3-6	metamorphosed tuff	pH = 7 (pure water)	90	28	4.78	3.82	0.26	0.00	46.67
WR6-3-7	metamorphosed tuff	pH = 7 (pure water)	90	35	4.76	3.48	0.12	0.00	47.58

Sample Number	Rock Type	Solution System	temperature	time	Na^+	K^+	Ca^{2+}	Mg^{2+}	H_2SiO_3
			℃	days	mg/L				
WR6-3-8	metamorphosed tuff	pH = 7 (pure water)	90	42	5.07	3.66	0.00	0.00	47.97
WR6-3-9	metamorphosed tuff	pH = 7 (pure water)	90	49	5.16	3.44	0.00	0.00	49.53
WR6-3-10	metamorphosed tuff	pH = 7 (pure water)	90	56	5.39	3.85	0.01	0.00	46.93
WR6-3-11	metamorphosed tuff	pH = 7 (pure water)	90	63	5.15	3.45	0.04	0.00	46.41
WR6-3-12	metamorphosed tuff	pH = 7 (pure water)	90	70	5.17	3.39	0.21	0.00	46.15
WR6-3-13	metamorphosed tuff	pH = 7 (pure water)	90	77	5.14	3.34	0.33	0.00	48.88
WR6-3-14	metamorphosed tuff	pH = 7 (pure water)	90	84	5.21	3.33	0.04	0.00	48.16
WR6-3-15	metamorphosed tuff	pH = 7 (pure water)	90	91	5.20	3.39	0.21	0.00	49.03
WR6-4-1	metamorphosed tuff	pH = 4 (HCl dilute solution)	60	1	2.30	4.20	1.64	0.17	10.01
WR6-4-2	metamorphosed tuff	pH = 4 (HCl dilute solution)	60	3	2.90	5.06	1.77	0.20	14.30
WR6-4-3	metamorphosed tuff	pH = 4 (HCl dilute solution)	60	7	3.18	5.74	2.15	0.25	20.28
WR6-4-4	metamorphosed tuff	pH = 4 (HCl dilute solution)	60	14	3.21	6.17	2.22	0.31	23.92
WR6-4-5	metamorphosed tuff	pH = 4 (HCl dilute solution)	60	21	3.88	5.97	2.02	0.00	23.92
WR6-4-6	metamorphosed tuff	pH = 4 (HCl dilute solution)	60	28	4.13	6.11	1.89	0.00	27.30
WR6-4-7	metamorphosed tuff	pH = 4 (HCl dilute solution)	60	35	4.25	5.97	1.80	0.00	28.99
WR6-4-8	metamorphosed tuff	pH = 4 (HCl dilute solution)	60	42	4.35	6.07	1.67	0.00	28.21
WR6-4-9	metamorphosed tuff	pH = 4 (HCl dilute solution)	60	49	4.25	5.52	1.48	0.00	29.38
WR6-4-10	metamorphosed tuff	pH = 4 (HCl dilute solution)	60	56	4.37	5.48	1.26	0.00	31.33
WR6-4-11	metamorphosed tuff	pH = 4 (HCl dilute solution)	60	63	4.51	5.36	1.44	0.00	31.20

Sample Number	Rock Type	Solution System	temperature	time	Na^+	K^+	Ca^{2+}	Mg^{2+}	H_2SiO_3
			℃	days			mg/L		
WR6-4-12	metamorphosed tuff	pH = 4 (HCl dilute solution)	60	70	4.56	5.17	1.53	0.00	31.72
WR6-4-13	metamorphosed tuff	pH = 4 (HCl dilute solution)	60	77	4.38	5.18	1.33	0.00	29.12
WR6-4-14	metamorphosed tuff	pH = 4 (HCl dilute solution)	60	84	4.49	4.91	1.31	0.00	31.59
WR6-4-15	metamorphosed tuff	pH = 4 (HCl dilute solution)	60	91	4.24	4.87	1.21	0.00	29.12
WR6-5-1	metamorphosed tuff	pH = 10 (NaOH dilute solution)	60	1	—	2.22	0.41	0.00	7.67
WR6-5-2	metamorphosed tuff	pH = 10 (NaOH dilute solution)	60	3	—	3.36	0.59	0.00	12.87
WR6-5-3	metamorphosed tuff	pH = 10 (NaOH dilute solution)	60	7	—	4.00	0.73	0.00	17.68
WR6-5-4	metamorphosed tuff	pH = 10 (NaOH dilute solution)	60	14	—	4.64	0.57	0.00	24.35
WR6-5-5	metamorphosed tuff	pH = 10 (NaOH dilute solution)	60	21	—	4.65	0.44	0.00	23.80
WR6-5-6	metamorphosed tuff	pH = 10 (NaOH dilute solution)	60	28	—	4.57	0.66	0.00	24.57
WR6-5-7	metamorphosed tuff	pH = 10 (NaOH dilute solution)	60	35	—	4.44	0.75	0.00	26.39
WR6-5-8	metamorphosed tuff	pH = 10 (NaOH dilute solution)	60	42	—	4.18	0.53	0.00	27.95
WR6-5-9	metamorphosed tuff	pH = 10 (NaOH dilute solution)	60	49	—	4.44	0.41	0.00	30.03
WR6-5-10	metamorphosed tuff	pH = 10 (NaOH dilute solution)	60	56	—	4.46	0.48	0.00	27.17
WR6-5-11	metamorphosed tuff	pH = 10 (NaOH dilute solution)	60	63	—	4.29	0.66	0.01	27.95
WR6-5-12	metamorphosed tuff	pH = 10 (NaOH dilute solution)	60	70	—	4.05	0.41	0.00	28.34
WR6-5-13	metamorphosed tuff	pH = 10 (NaOH dilute solution)	60	77	—	3.85	0.84	0.00	28.21
WR6-5-14	metamorphosed tuff	pH = 10 (NaOH dilute solution)	60	84	—	3.66	1.04	0.00	27.95
WR6-5-15	metamorphosed tuff	pH = 10 (NaOH dilute solution)	60	91	—	3.68	0.97	0.00	29.77

Sample Number	Rock Type	Solution System	temperature	time	Na^+	K^+	Ca^{2+}	Mg^{2+}	H_2SiO_3
			℃	days	mg/L				
WR6-6-1	metamorphosed quartz sandstone	pH = 7 (pure water)	25	1	2.34	1.88	5.09	0.19	3.90
WR6-6-2	metamorphosed quartz sandstone	pH = 7 (pure water)	25	3	2.47	2.13	5.21	0.20	4.94
WR6-6-3	metamorphosed quartz sandstone	pH = 7 (pure water)	25	7	2.90	2.28	5.35	0.26	4.68
WR6-6-4	metamorphosed quartz sandstone	pH = 7 (pure water)	25	14	2.95	2.35	5.84	0.28	5.72
WR6-6-5	metamorphosed quartz sandstone	pH = 7 (pure water)	25	21	3.28	2.44	6.28	0.33	5.33
WR6-6-6	metamorphosed quartz sandstone	pH = 7 (pure water)	25	28	3.38	2.44	6.00	0.35	5.98
WR6-6-7	metamorphosed quartz sandstone	pH = 7 (pure water)	25	35	3.27	2.41	6.47	0.36	5.59
WR6-6-8	metamorphosed quartz sandstone	pH = 7 (pure water)	25	42	3.30	2.39	7.07	0.35	5.46
WR6-6-9	metamorphosed quartz sandstone	pH = 7 (pure water)	25	49	3.25	2.35	7.68	0.44	5.46
WR6-6-10	metamorphosed quartz sandstone	pH = 7 (pure water)	25	56	3.39	2.43	7.72	0.44	5.85
WR6-6-11	metamorphosed quartz sandstone	pH = 7 (pure water)	25	63	3.50	2.39	8.13	0.50	5.72
WR6-6-12	metamorphosed quartz sandstone	pH = 7 (pure water)	25	70	3.33	2.42	8.30	0.49	6.28
WR6-6-13	metamorphosed quartz sandstone	pH = 7 (pure water)	25	77	3.15	2.40	8.45	0.49	5.07
WR6-6-14	metamorphosed quartz sandstone	pH = 7 (pure water)	25	84	2.82	2.42	8.97	0.52	6.67
WR6-6-15	metamorphosed quartz sandstone	pH = 7 (pure water)	25	91	2.93	2.40	9.02	0.54	5.33
WR6-7-1	metamorphosed quartz sandstone	pH = 7 (pure water)	60	1	4.04	3.19	6.52	0.18	12.35
WR6-7-2	metamorphosed quartz sandstone	pH = 7 (pure water)	60	3	4.77	3.39	7.47	0.11	15.98
WR6-7-3	metamorphosed quartz sandstone	pH = 7 (pure water)	60	7	5.27	3.49	8.74	0.17	16.64
WR6-7-4	metamorphosed quartz sandstone	pH = 7 (pure water)	60	14	5.57	3.49	11.52	0.32	16.90

Sample Number	Rock Type	Solution System	temperature	time	Na^{+}	K^{+}	Ca^{2+}	Mg^{2+}	H_2SiO_3
			℃	days	mg/L				
WR6-7-5	metamorphosed quartz sandstone	pH = 7 (pure water)	60	21	5.90	3.50	12.77	0.38	17.16
WR6-7-6	metamorphosed quartz sandstone	pH = 7 (pure water)	60	28	5.96	3.48	13.52	0.42	18.07
WR6-7-7	metamorphosed quartz sandstone	pH = 7 (pure water)	60	35	6.04	3.58	14.06	0.44	20.28
WR6-7-8	metamorphosed quartz sandstone	pH = 7 (pure water)	60	42	6.13	3.56	14.09	0.46	20.02
WR6-7-9	metamorphosed quartz sandstone	pH = 7 (pure water)	60	49	5.99	3.59	14.43	0.49	19.63
WR6-7-10	metamorphosed quartz sandstone	pH = 7 (pure water)	60	56	6.08	3.64	14.43	0.51	20.15
WR6-7-11	metamorphosed quartz sandstone	pH = 7 (pure water)	60	63	6.16	3.59	14.39	0.48	23.14
WR6-7-12	metamorphosed quartz sandstone	pH = 7 (pure water)	60	70	6.08	3.59	14.05	0.48	21.59
WR6-7-13	metamorphosed quartz sandstone	pH = 7 (pure water)	60	77	6.08	3.51	13.81	0.47	23.01
WR6-7-14	metamorphosed quartz sandstone	pH = 7 (pure water)	60	84	6.16	3.49	13.14	0.41	22.49
WR6-7-15	metamorphosed quartz sandstone	pH = 7 (pure water)	60	91	6.19	3.50	13.20	0.38	23.35
WR6-8-1	metamorphosed quartz sandstone	pH = 7 (pure water)	90	1	5.56	3.40	6.86	0.00	20.80
WR6-8-2	metamorphosed quartz sandstone	pH = 7 (pure water)	90	3	6.81	3.98	6.73	0.00	24.83
WR6-8-3	metamorphosed quartz sandstone	pH = 7 (pure water)	90	7	7.49	4.08	6.76	0.00	27.43
WR6-8-4	metamorphosed quartz sandstone	pH = 7 (pure water)	90	14	7.74	4.23	6.81	0.00	31.59
WR6-8-5	metamorphosed quartz sandstone	pH = 7 (pure water)	90	21	7.95	4.25	7.20	0.00	30.03
WR6-8-6	metamorphosed quartz sandstone	pH = 7 (pure water)	90	28	8.13	4.36	7.86	0.00	33.54
WR6-8-7	metamorphosed quartz sandstone	pH = 7 (pure water)	90	35	7.92	4.18	8.03	0.00	32.37
WR6-8-8	metamorphosed quartz sandstone	pH = 7 (pure water)	90	42	7.96	4.07	7.91	0.00	32.24

Sample Number	Rock Type	Solution System	temperature	time	Na^{+}	K^{+}	Ca^{2+}	Mg^{2+}	H_2SiO_3
			℃	days	mg/L				
WR6-8-9	metamorphosed quartz sandstone	pH = 7 (pure water)	90	49	7.96	4.07	8.05	0.00	34.32
WR6-8-10	metamorphosed quartz sandstone	pH = 7 (pure water)	90	56	7.91	3.96	7.83	0.00	34.71
WR6-8-11	metamorphosed quartz sandstone	pH = 7 (pure water)	90	63	7.72	3.96	7.91	0.00	32.76
WR6-8-12	metamorphosed quartz sandstone	pH = 7 (pure water)	90	70	7.65	3.94	8.06	0.00	30.94
WR6-8-13	metamorphosed quartz sandstone	pH = 7 (pure water)	90	77	7.47	3.92	8.03	0.00	33.41
WR6-8-14	metamorphosed quartz sandstone	pH = 7 (pure water)	90	84	7.32	4.05	8.15	0.00	33.28
WR6-8-15	metamorphosed quartz sandstone	pH = 7 (pure water)	90	91	7.31	3.73	7.97	0.00	35.92
WR6-9-1	metamorphosed quartz sandstone	pH = 4 (HCl dilute solution)	60	1	3.23	2.31	7.95	0.17	8.45
WR6-9-2	metamorphosed quartz sandstone	pH = 4 (HCl dilute solution)	60	3	4.56	2.78	9.31	0.21	10.66
WR6-9-3	metamorphosed quartz sandstone	pH = 4 (HCl dilute solution)	60	7	5.39	3.44	12.27	0.36	14.43
WR6-9-4	metamorphosed quartz sandstone	pH = 4 (HCl dilute solution)	60	14	5.58	3.06	13.41	0.41	16.12
WR6-9-5	metamorphosed quartz sandstone	pH = 4 (HCl dilute solution)	60	21	5.59	3.18	15.12	0.52	17.81
WR6-9-6	metamorphosed quartz sandstone	pH = 4 (HCl dilute solution)	60	28	5.63	3.07	14.87	0.45	18.20
WR6-9-7	metamorphosed quartz sandstone	pH = 4 (HCl dilute solution)	60	35	5.71	3.07	15.56	0.46	19.37
WR6-9-8	metamorphosed quartz sandstone	pH = 4 (HCl dilute solution)	60	42	6.11	2.84	15.56	0.47	19.11
WR6-9-9	metamorphosed quartz sandstone	pH = 4 (HCl dilute solution)	60	49	5.87	3.06	15.98	0.51	20.93
WR6-9-10	metamorphosed quartz sandstone	pH = 4 (HCl dilute solution)	60	56	6.50	3.06	15.98	0.49	20.93
WR6-9-11	metamorphosed quartz sandstone	pH = 4 (HCl dilute solution)	60	63	5.92	2.87	15.80	0.56	19.59
WR6-9-12	metamorphosed quartz sandstone	pH = 4 (HCl dilute solution)	60	70	5.73	2.80	15.49	0.54	20.81

Sample Number	Rock Type	Solution System	temperature	time	Na^{+}	K^{+}	Ca^{2+}	Mg^{2+}	H_2SiO_3
			℃	days	mg/L				
WR6-9-13	metamorphosed quartz sandstone	pH = 4 (HCl dilute solution)	60	77	5.77	2.94	15.46	0.57	22.49
WR6-9-14	metamorphosed quartz sandstone	pH = 4 (HCl dilute solution)	60	84	5.83	3.09	15.03	0.56	20.37
WR6-9-15	metamorphosed quartz sandstone	pH = 4 (HCl dilute solution)	60	91	6.15	2.78	15.01	0.58	23.01
WR6-10-1	metamorphosed quartz sandstone	pH = 10 (NaOH dilute solution)	60	1	—	2.71	4.98	0.00	13.52
WR6-10-2	metamorphosed quartz sandstone	pH = 10 (NaOH dilute solution)	60	3	—	3.06	5.28	0.00	15.86
WR6-10-3	metamorphosed quartz sandstone	pH = 10 (NaOH dilute solution)	60	7	—	2.89	5.29	0.00	17.03
WR6-10-4	metamorphosed quartz sandstone	pH = 10 (NaOH dilute solution)	60	14	—	2.95	5.53	0.00	16.77
WR6-10-5	metamorphosed quartz sandstone	pH = 10 (NaOH dilute solution)	60	21	—	3.08	7.73	0.00	18.07
WR6-10-6	metamorphosed quartz sandstone	pH = 10 (NaOH dilute solution)	60	28	—	3.00	9.52	0.14	17.81
WR6-10-7	metamorphosed quartz sandstone	pH = 10 (NaOH dilute solution)	60	35	—	2.88	10.16	0.18	17.94
WR6-10-8	metamorphosed quartz sandstone	pH = 10 (NaOH dilute solution)	60	42	—	3.04	11.45	0.30	18.33
WR6-10-9	metamorphosed quartz sandstone	pH = 10 (NaOH dilute solution)	60	49	—	2.82	11.84	0.29	18.59
WR6-10-10	metamorphosed quartz sandstone	pH = 10 (NaOH dilute solution)	60	56	—	3.11	12.31	0.34	19.89
WR6-10-11	metamorphosed quartz sandstone	pH = 10 (NaOH dilute solution)	60	63	—	2.94	11.74	0.34	21.45
WR6-10-12	metamorphosed quartz sandstone	pH = 10 (NaOH dilute solution)	60	70	—	2.87	13.14	0.42	20.41
WR6-10-13	metamorphosed quartz sandstone	pH = 10 (NaOH dilute solution)	60	77	—	2.81	13.14	0.45	20.41
WR6-10-14	metamorphosed quartz sandstone	pH = 10 (NaOH dilute solution)	60	84	—	2.81	13.11	0.41	20.80
WR6-10-15	metamorphosed quartz sandstone	pH = 10 (NaOH dilute solution)	60	91	—	2.63	13.10	0.43	21.19
WR6-11-1	slate	pH = 7 (pure water)	25	1	0.83	1.28	4.80	0.21	2.99

Sample Number	Rock Type	Solution System	temperature	time	Na^+	K^+	Ca^{2+}	Mg^{2+}	H_2SiO_3
			℃	days	mg/L				
WR6-11-2	slate	pH = 7 (pure water)	25	3	0.86	1.43	5.04	0.24	3.43
WR6-11-3	slate	pH = 7 (pure water)	25	7	1.48	1.75	5.46	0.34	3.64
WR6-11-4	slate	pH = 7 (pure water)	25	14	1.45	1.81	5.51	0.34	3.82
WR6-11-5	slate	pH = 7 (pure water)	25	21	1.57	1.89	6.24	0.44	4.16
WR6-11-6	slate	pH = 7 (pure water)	25	28	1.55	1.82	6.67	0.59	4.25
WR6-11-7	slate	pH = 7 (pure water)	25	35	1.54	1.87	6.78	0.53	5.33
WR6-11-8	slate	pH = 7 (pure water)	25	42	1.52	1.96	7.23	0.63	4.77
WR6-11-9	slate	pH = 7 (pure water)	25	49	1.21	2.10	7.51	0.60	4.81
WR6-11-10	slate	pH = 7 (pure water)	25	56	1.27	2.08	7.72	0.63	4.03
WR6-11-11	slate	pH = 7 (pure water)	25	63	1.39	1.90	7.96	0.65	5.85
WR6-11-12	slate	pH = 7 (pure water)	25	70	1.36	2.03	8.29	0.67	4.94
WR6-11-13	slate	pH = 7 (pure water)	25	77	1.61	1.97	8.30	0.66	5.46
WR6-11-14	slate	pH = 7 (pure water)	25	84	1.57	2.01	8.59	0.69	4.29
WR6-11-15	slate	pH = 7 (pure water)	25	91	1.62	2.01	8.57	0.67	5.85
WR6-12-1	slate	pH = 7 (pure water)	60	1	1.56	2.13	5.76	0.19	4.68
WR6-12-2	slate	pH = 7 (pure water)	60	3	2.24	3.09	7.82	0.30	8.32
WR6-12-3	slate	pH = 7 (pure water)	60	7	2.71	3.89	10.64	0.48	10.14
WR6-12-4	slate	pH = 7 (pure water)	60	14	3.03	4.25	12.03	0.50	14.30
WR6-12-5	slate	pH = 7 (pure water)	60	21	2.91	4.08	12.42	0.51	12.22

Sample Number	Rock Type	Solution System	temperature	time	Na^+	K^+	Ca^{2+}	Mg^{2+}	H_2SiO_3
			℃	days	mg/L				
WR6-12-6	slate	pH = 7 (pure water)	60	28	3.17	4.13	12.64	0.49	13.52
WR6-12-7	slate	pH = 7 (pure water)	60	35	3.21	4.05	12.82	0.48	13.26
WR6-12-8	slate	pH = 7 (pure water)	60	42	3.56	4.09	12.94	0.47	13.26
WR6-12-9	slate	pH = 7 (pure water)	60	49	3.54	4.19	13.37	0.45	13.78
WR6-12-10	slate	pH = 7 (pure water)	60	56	3.51	3.84	13.11	0.45	13.52
WR6-12-11	slate	pH = 7 (pure water)	60	63	2.98	3.90	13.03	0.41	13.26
WR6-12-12	slate	pH = 7 (pure water)	60	70	3.49	3.88	13.10	0.40	14.82
WR6-12-13	slate	pH = 7 (pure water)	60	77	3.10	3.82	12.89	0.36	14.74
WR6-12-14	slate	pH = 7 (pure water)	60	84	3.21	3.59	12.78	0.35	15.21
WR6-12-15	slate	pH = 7 (pure water)	60	91	2.96	3.67	12.51	0.29	14.04
WR6-13-1	slate	pH = 7 (pure water)	90	1	2.10	3.42	6.36	0.09	12.09
WR6-13-2	slate	pH = 7 (pure water)	90	3	3.19	4.71	7.82	0.04	16.12
WR6-13-3	slate	pH = 7 (pure water)	90	7	3.69	4.49	8.70	0.15	19.37
WR6-13-4	slate	pH = 7 (pure water)	90	14	4.49	4.37	8.91	0.16	19.37
WR6-13-5	slate	pH = 7 (pure water)	90	21	4.82	4.11	9.00	0.00	22.49
WR6-13-6	slate	pH = 7 (pure water)	90	28	5.35	3.86	8.53	0.00	24.31
WR6-13-7	slate	pH = 7 (pure water)	90	35	5.28	3.83	8.39	0.00	24.57
WR6-13-8	slate	pH = 7 (pure water)	90	42	5.47	3.73	8.21	0.00	26.36
WR6-13-9	slate	pH = 7 (pure water)	90	49	5.64	3.44	7.67	0.00	27.04

Sample Number	Rock Type	Solution System	temperature	time	Na^+	K^+	Ca^{2+}	Mg^{2+}	H_2SiO_3
			℃	days	mg/L				
WR6-13-10	slate	pH = 7 (pure water)	90	56	5.67	3.70	7.67	0.00	26.65
WR6-13-11	slate	pH = 7 (pure water)	90	63	5.69	3.14	7.79	0.00	24.44
WR6-13-12	slate	pH = 7 (pure water)	90	70	5.82	3.19	7.97	0.00	26.13
WR6-13-13	slate	pH = 7 (pure water)	90	77	5.58	3.21	7.96	0.00	26.91
WR6-13-14	slate	pH = 7 (pure water)	90	84	5.45	3.06	7.87	0.00	26.36
WR6-13-15	slate	pH = 7 (pure water)	90	91	5.34	3.27	7.79	0.00	26.92
WR6-14-1	slate	pH = 4 (HCl dilute solution)	60	1	1.80	3.05	7.58	0.40	6.24
WR6-14-2	slate	pH = 4 (HCl dilute solution)	60	3	2.42	3.75	9.45	0.50	8.19
WR6-14-3	slate	pH = 4 (HCl dilute solution)	60	7	2.52	4.28	12.32	0.71	9.75
WR6-14-4	slate	pH = 4 (HCl dilute solution)	60	14	2.82	4.52	13.34	0.74	12.74
WR6-14-5	slate	pH = 4 (HCl dilute solution)	60	21	2.84	4.32	14.06	0.84	12.09
WR6-14-6	slate	pH = 4 (HCl dilute solution)	60	28	2.88	4.39	14.46	0.75	13.91
WR6-14-7	slate	pH = 4 (HCl dilute solution)	60	35	2.87	4.33	14.55	0.72	13.65
WR6-14-8	slate	pH = 4 (HCl dilute solution)	60	42	2.86	4.09	14.90	0.71	13.26
WR6-14-9	slate	pH = 4 (HCl dilute solution)	60	49	3.03	4.16	14.44	0.69	12.22
WR6-14-10	slate	pH = 4 (HCl dilute solution)	60	56	3.01	4.24	14.70	0.65	13.99
WR6-14-11	slate	pH = 4 (HCl dilute solution)	60	63	3.01	4.12	14.83	0.65	14.04
WR6-14-12	slate	pH = 4 (HCl dilute solution)	60	70	3.07	4.03	14.46	0.70	13.65
WR6-14-13	slate	pH = 4 (HCl dilute solution)	60	77	2.98	4.04	14.75	0.64	12.61

Sample Number	Rock Type	Solution System	temperature	time	Na^+	K^+	Ca^{2+}	Mg^{2+}	H_2SiO_3
			℃	days	mg/L				
WR6-14-14	slate	pH = 4 (HCl dilute solution)	60	84	2.98	3.76	14.38	0.60	13.00
WR6-14-15	slate	pH = 4 (HCl dilute solution)	60	91	2.92	3.80	14.06	0.54	12.92
WR6-15-1	slate	pH = 10 (NaOH dilute solution)	60	1	—	1.94	5.42	0.04	5.33
WR6-15-2	slate	pH = 10 (NaOH dilute solution)	60	3	—	3.19	7.08	0.11	8.58
WR6-15-3	slate	pH = 10 (NaOH dilute solution)	60	7	—	3.64	9.20	0.26	10.66
WR6-15-4	slate	pH = 10 (NaOH dilute solution)	60	14	—	3.76	10.19	0.29	11.05
WR6-15-5	slate	pH = 10 (NaOH dilute solution)	60	21	—	3.82	10.77	0.28	12.74
WR6-15-6	slate	pH = 10 (NaOH dilute solution)	60	28	—	3.77	11.27	0.29	13.91
WR6-15-7	slate	pH = 10 (NaOH dilute solution)	60	35	—	3.82	11.59	0.28	13.39
WR6-15-8	slate	pH = 10 (NaOH dilute solution)	60	42	—	3.55	11.75	0.30	13.13
WR6-15-9	slate	pH = 10 (NaOH dilute solution)	60	49	—	3.61	10.82	0.23	14.43
WR6-15-10	slate	pH = 10 (NaOH dilute solution)	60	56	—	3.42	10.90	0.24	12.22
WR6-15-11	slate	pH = 10 (NaOH dilute solution)	60	63	—	3.38	11.50	0.25	12.22
WR6-15-12	slate	pH = 10 (NaOH dilute solution)	60	70	—	3.35	11.63	0.22	11.44
WR6-15-13	slate	pH = 10 (NaOH dilute solution)	60	77	—	3.24	11.70	0.23	13.26
WR6-15-14	slate	pH = 10 (NaOH dilute solution)	60	84	—	3.24	11.94	0.26	11.44
WR6-15-15	slate	pH = 10 (NaOH dilute solution)	60	91	—	3.22	11.98	0.22	12.79
